# A Comparison of the Effects of Continuous Illumination and Day/Night Regimes on PHB Accumulation in *Synechocystis* Cells

**DOI:** 10.3390/life14070907

**Published:** 2024-07-20

**Authors:** Christina Fleischhacker-Daffert, Antonia Zerobin, Ferdinand Hummel, Eva Slaninova, Zuzana Kroupová, Stanislav Obruca, Katerina Mrazova, Kamila Hrubanova, Vladislav Krzyzanek, Jana Nebesarova, Katharina Ludwig, Ines Fritz

**Affiliations:** 1 Institute of Environmental Biotechnology, Department of Agrobiotechnology, IFA-Tulln, University of Natural Resources and Life Sciences, Vienna, Konrad-Lorenz Straße 20, 3430 Tulln, Austria; christina.daffert@boku.ac.at (C.F.-D.); ferdinand.hummel@boku.ac.at (F.H.); ines.fritz@boku.ac.at (I.F.); 2 Department of Food Chemistry and Biotechnology, Faculty of Chemistry, Brno University of Technology, Purkynova 118, 61200 Brno, Czech Republic; xcslaninovae@fch.vut.cz (E.S.); zuzana.kroupova@vut.cz (Z.K.); obruca@fch.vut.cz (S.O.); mrazova@isibrno.cz (K.M.); 3 Institute of Scientific Instruments, The Czech Academy of Sciences, Královopolská 147, 61264 Brno, Czech Republic; hrubanova@isibrno.cz (K.H.); krzyzanek@isibrno.cz (V.K.); 4 Institute of Parasitology, Biology Centre, The Czech Academy of Sciences, Branisovska 31, 37005 Ceske Budejovice, Czech Republic; nebe@paru.cas.cz; 5 Faculty of Science, Charles University, Vinicna 7, 12844 Prague, Czech Republic; 6 BEST—Bioenergy and Sustainable Technologies GmbH, Inffeldgasse 21b, 8010 Graz, Austria

**Keywords:** *Synechocystis*, continuous illumination, day/night cycle, PHB, glycogen, cell size

## Abstract

Poly(3-hydroxybutyrate) (PHB) is a biobased and biodegradable polymer with properties comparable to polypropylene and therefore has the potential to replace conventional plastics. PHB is intracellularly accumulated by prokaryotic organisms. For the cells PHB functions manly as carbon and energy source, but all possible functions of PHB are still not known. *Synechocystis* (cyanobacteria) accumulates PHB using light as energy and CO_2_ as carbon source. The main trigger for PHB accumulation in cyanobacteria is nitrogen and phosphorous depletion with simultaneous surplus of carbon and energy. For the above reasons, obtaining knowledge about external factors influencing PHB accumulation is of highest interest. This study compares the effect of continuous light exposure and day/night (16/8 h) cycles on selected physiology parameters of three *Synechocystis* strains. We show that continuous illumination at moderate light intensities leads to an increased PHB accumulation in *Synechocystis salina* CCALA 192 (max. 14.2% CDW – cell dry weight) compared to day/night cycles (3.7% CDW). In addition to PHB content, glycogen and cell size increased, while cell density and cell viability decreased. The results offer new approaches for further studies to gain deeper insights into the role of PHB in cyanobacteria to obtain bioplastics in a more sustainable and environmentally friendly way.

## 1. Introduction

Cyanobacteria are photoautotrophic organisms that can produce technologically interesting biomolecules such as polyhydroxybutyrate (PHB), which belongs to the polyhydroxyalkanoates (PHA), by using CO_2_ as carbon and light as an energy source [1,2]. PHB in general is industrially valuable as it is a biodegradable biopolymer with comparable properties to polypropylene and therefore has the potential to replace conventional plastics [3]. 

Industrial PHB production is currently based on heterotrophic organisms due to their high accumulation rates [4]. However, these use agricultural products as their carbon source. This conflict could be avoided with the photoautotrophic production of PHB with cyanobacteria, as cyanobacteria utilize CO_2_ (e.g., from the ambient air or industrial off-gases) [5]. Unfortunately, they still have a major drawback because the PHB content, which is achieved with photoautotrophic cultivation, is most often below 10% of cell dry weight (CDW), sometimes even below 2% CDW [1]. In comparison, in heterotrophic cells polymer concentrations typically exceed 75% CDW [5]. Additionally, cell densities and cell growth in phototrophic systems are lower than in heterotrophic processes [5]. Due to these reasons, it is important to gain more information about PHB accumulation and options to increase the PHB content in cyanobacteria under photoautotrophic conditions.

Cyanobacteria belong to the very diverse group of organisms, which include more than 6000 species ranging from a size of 1 µm (*Synechocystis* sp.) to the several millimeter-long multicellular *Oscillatoria* sp. [6]. These organisms can live in a wide range of ecological habitats due to their ability to adapt to changing environmental conditions and stress factors, such as UV exposure, temperature, salt, and metals by adjusting physiological, biochemical, and molecular activities [7,8]. When growth is limited by nitrogen and phosphorous, some cyanobacteria increasingly synthesize glycogen (a polymer consisting of glucose monomers) and PHB as storage compounds. Glycogen is consumed in the dark phases to regulate energy availability between dark and light periods, whereas PHB mainly serves as an electron sink and as a long-term carbon store [2,9]. It was reported that glycogen and PHB metabolism play an important role in the regulation of energy charge in the cells by ATP (adenosine triphosphate) and NADPH (nicotinamide adenine dinucleotide phosphate) homeostasis, respectively [10,11]. In addition, it is known that an increased PHB content in the cell can help to better compensate for various stress factors in heterotrophic bacteria [12]. Also possible is the stimulation of PHB production through mild stress conditions, while the main trigger of PHB accumulation in cyanobacteria is nitrogen and phosphorous depletion and the simultaneous excess of carbon and energy [8,13]. However, all the functions of PHB in the cell are not yet fully explored and known [2,14]. In contrast to glycogen, PHB does not appear to be necessary for cyanobacteria to survive. Even mutants in which PHB synthesis is suppressed can reactivate from chlorosis [13].

In general, cyanobacteria convert light energy into chemical energy by absorbing light within the photosynthetically active radiation (PAR) between 400 and 700 nm for photosynthesis through their photoactive pigments [1]. When growing in a natural environment, cyanobacteria are exposed to day/night cycles and thereby respond with an endogenous circadian clock that regulates gene expression [15,16]. Excess light energy creates reactive oxygen species (ROS), which in turn can lead to the inhibition of protein synthesis and can also induce photoinhibition [17]. Photoinhibition is referred to as the inactivation of photosystem II (PSII), a pigment complex responsible for converting light energy [18]. As photosynthetic organisms depend on light for photosynthesis, they can be very sensitive to changes in light conditions and can cause different adaptions in growth, development, or metabolism [19], but also, morphological adjustments in photosynthetic organisms can be caused [17]. Many research groups investigated the oxidative stress of extensive light in the form of high intensities or UV-irradiation, while no studies were found that addressed the effect of continuous light, even though they are often grown under continuous light as a standard cultivation condition in the laboratory [20].

There are only a few publications dealing with the effect of light on PHB production in cyanobacteria. Table 1 displays studies in which the effect of different light regimes on PHB production in cyanobacteria were investigated. All authors measured either no significant difference or even higher PHB accumulation at day/night cycles compared to continuous illumination. No reports were found that focused on light stress from continuous illumination and its effect on PHB accumulation.

This study aims to investigate how continuous illumination affects growth and the accumulation of storage compounds in *Synechocystis*. It compares the response of three unicellular *Synechocystis* strains to continuous light exposure and day/night (16/8 h) cycles on growth, the accumulation of PHB and glycogen, cell size, and viability. *Synechocystis* PCC 6803 is one of the best-studied cyanobacterial strains and serves as a reference to existing research. *Synechocystis* CCALA 192 has proven to be a promising PHB producer after the screening of around 30 strains and is also easy to cultivate [6,25]. *Synechocystis* IFA-3 was isolated, identified (16S rDNA), and proven as PHB producer [26].

## 2. Materials and Methods

### 2.1. Strains

In this study, three *Synechocystis* strains were used, namely *S. salina* CCALA192 (hereafter Syn.192), which was obtained from the culture collection of autotrophic organisms (CZE); *Synechocstis* sp. PCC6803 (hereafter Syn.6803) from Pasteur culture collection (FRA); and *Synechocystis* IFA-3 (hereafter IFA-3), which is a wild-type cyanobacteria, isolated in Tulln (AUT) [26]. 

All strains were cultivated in an adjusted BG-11 Medium [27] with changed nitrogen and phosphorous concentrations to induce nitrogen limitation automatically [8].

### 2.2. Experimental Setup

The strains were cultivated in 500 mL Erlenmeyer flasks in a starting volume of 200 mL adjusted BG-11 medium. Due to sampling, volume decreased over the cultivation time. Exactly the same volume was taken from all flasks at each sampling; therefore, a possible effect of reduced volume on the cultures would occur equally in all flasks. Taking a sample without refilling the volume afterwards guaranties that there is no dilution of the cultures and no change in the original media composition that is not caused exclusively by the cells.

**Figure 1:**
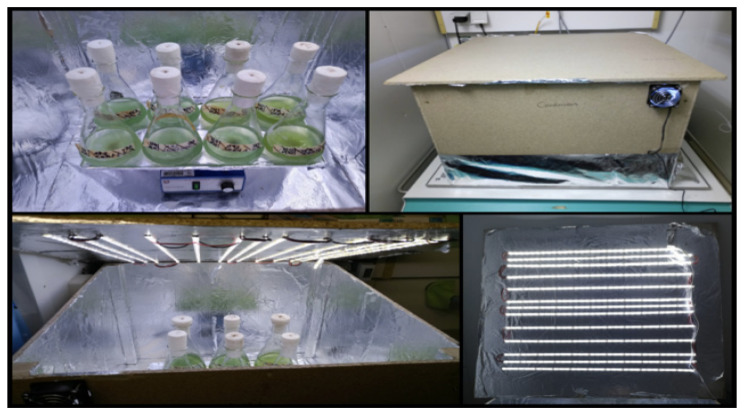
The set-up of the incubation boxes for cultivation with continuous illumination and day/night cycles; (**top left**): flasks on a shaker inside the cultivation box; (**top right**): one of the two used cultivation boxes from the outside with an installed computer fan; (**bottom left**): the view into the cultivation box during the running experiment; (**bottom right**): the LED strip arrangement.

To ensure different illumination times (continuous illumination and day/night (16/8 h) cycles) but identical environmental conditions, two light-tight cultivation boxes were built, which were illuminated with LEDs (SimpLED, 6500 K, 12 V, 450LED, Paulmann Licht GmbH, Springe-Völksen, Germany). The specific construction (Figure 1) led to a light intensity of 86 µmol photons m^−2^ s^−1^. At the beginning of the experiment, the light intensity was measured at three different points on the shaking platform at the positions of the flasks in the box using a light meter [µmol photons m^−2^s^−1^] (ULM-500, Heinz Walz GmbH, Effeltrich, Germany) and averaged.

These boxes were placed in an air-conditioned room with a temperature of 20 °C and ambient CO_2_ concentration. Illumination led to temperatures of 25 °C inside the boxes. This temperature was monitored continuously with a data logger (HOBO, Pendant, MX Temp/Light, Onset, Bourne, MA, USA). A computer fan was installed in the box to prevent overheating. The flasks were placed on a shaker on the inside. The walls were wrapped with aluminum foil to ensure the reflection of light. After each sampling, the flasks were randomly placed back on the shaker to compensate for small differences between the respective positions.

Similar culture conditions described in materials and methods were obtained, except for the illumination time. The experiments for Syn.192 were performed in a 9-fold replicate, Syn.6803 6-fold, and IFA-3 3-fold. Since this was not possible in one experimental run, the experiments were performed in 3 sets and then tested for normal distribution using the Shapiro–Wilk test to determine whether data from one measurement day may be combined. This was the case for each measurement day. The cultivation period of the experiments were 40 days.

### 2.3. Growth Monitoring

Before sampling, evaporated water was replaced on weight basis.

Growth was monitored with optical density at 750 nm (OD_750_) twice a week. Once a week, cell dry weight (CDW) [g L^−1^] was determined as part of the analysis of cell components. For this purpose, 10 mL of sample were taken once a week and dried at 105 °C.

On the last day of cultivation, the cell count of each flask was determined with a cell counting chamber (Neubauer Improved). The nitrate level was determined with a HACH kit (LCK339, Hach Lange GmbH, Vienna, Austria) with automated barcode detection and analysis by a HACH photometer (Dr2800, Hach Lange GmbH, Vienna, Austria) to evaluate whether the cultures reached nitrogen starvation.

### 2.4. PHB Analysis

Based on Karr et al. (1983) [28], 200 µL of 97% H_2_SO_4_ was added to the dried cell pellet for cell disruption and conversion of PHB into crotonic acid, which was quantified with HPLC (high-performance liquid chromatography). The pellets were resuspended in an ultrasonic bath and afterwards incubated for 30 min at 90 °C. After cooling down to room temperature, 9.8 mL of Milli-Q^®^ water was added, homogenized, and filtrated through a 0.45 µm polyamide syringe filter before HPLC analysis. Crotonic acid was determined with an Agilent 1100 system (Agilent Technologies, Inc., Santa Clara, CA, USA) with an ion exchange column (CARBOSep Coregel 87H, part no. CHO-99-9861, 0.01 N H_2_SO_4_). In addition, a second determination with a smaller sample volume (5 mL) was carried out.

### 2.5. Glycogen Analysis

The method for determination of glycogen content was adapted from Koch et al. (2019) [13]. An amount of 2 mL of culture was harvested and centrifuged at 8000 rpm to remove the supernatant. The pellet was washed twice with double-distilled water and afterwards resuspended with 400 µL KOH (30% (*w*/*v*)) to destroy cell walls. Afterwards, the mixture was incubated for 2 h at 95 °C and 450 rpm. An amount of 1200 µL ice-cold 98% ethanol was added for glycogen precipitation. The samples were then centrifuged at 10,000× *g* for 10 min at 4 °C. Two washing steps with 700 µL ethanol followed (1st with 70% (*v*/*v*) ethanol and 2nd with absolute ethanol) for removal of KOH, with a final centrifugation step. The pellet was dried at 60 °C. For detection, glycogen was converted into glucose by adding 1 mL of 100 mM sodium acetate (pH 4.5) and 10 µL (0.62 U µL^−1^) of amyloglucosidase solution to the pellet and incubation at 55 °C and 450 rpm for 2 h. After Carrez precipitation (Carrez solution I: 106.5 g L^−1^ K_4_[Fe(CN)_6_] × 3H_2_O; Carrez Solution II: 288 g L^−1^ ZnSO_4_ × 7H_2_O), the solution was filtered through a syringe filter (0.45 µm) for HPLC (Agilent 1100, ICSep ICE-ION-300, Transgenomic, refractive index detector, 0.01 N H_2_SO_4_). Glycogen (≥85% (enzyme) from oyster, Honeywell Fluka^TM^, Seelze, Germany) was treated in the same way as the sample to determine the method’s recovery rate.

### 2.6. Chlorophyll_a_ Analysis

When analyzing the chlorophyll_a_ content, the washed wet biomass pellet was extracted with ethanol (absolute) (adapted from Zavrel et al. (2015) [29]. The pellet was stored for 2 days at 4 °C to extract chlorophyll_a_. Absorbances of the extract were measured with a UV–Vis spectrometer (UV-1800, Shimadzu Duisburg, Germany) at 665 nm and calculated with Equation (1) [30].

(1) $${Chlorophyll}_{a}\;[{mg\;L}^{-1}]={13.2969*A}_{665\;nm}$$

### 2.7. Flow Cytometer

Determination of the cell viability of *Synechocystis* strains was performed with flow cytometry using fluorescent dye SYTOX™ Blue Dead Cell Stain (1 mM solution in DMSO, Fisher Scientific GmbH, Schwerte, Germany) as a viability probe. At first, cyanobacterial cultures were washed with PBS buffer (pH 7.4), then resuspended and diluted by the buffer to a cell density of approx. 10^6^ cells per mL. After that, 1 μL of SYTOX™ Blue Dead Cell Stain was added to the suspensions (the final concentration of the stain in 1 mL of the sample was 1 μM) and then bacterial suspensions were incubated in the dark at laboratory temperature for 5 min. After incubation, the stained and non-stained samples were immediately measured at single cell level using the flow cytometer Cytek Aurora (Cytek Biosciences Inc. Amsterdam, The Netherlands), and the signal was observed in collecting channel V4 (473 ± 8 nm).

### 2.8. Confocal Microscopy/Measurement of Cell Size

Cell suspensions from the last day of cultivation were used for PHB colorization and visualization with a confocal scanning microscope. An amount of 100 µL (the extracted cell suspension was diluted to an applicable cell density) were treated with 11 µL Nile-red solution (50 µg mL^−1^ in 98% ethanol). An amount of 20 µL of colored cell suspension was dried and finally heat fixated on microscopic slides by passing the slide through a flame very quickly. The samples were analyzed with a confocal scanning microscope (Leica Microsystems, TCS II SP5, Mannheim, Germany; Objective: HCX PL APO CS 63 × 1.20 water immersion) and irradiated with an argon laser at 488 nm. Detection was obtained with the photomultiplier tube PMT3 with the Leica/EGFP setting at an emission bandwidth of 548 nm to 596 nm. Pictures were processed, and cell size was measured by determining the cell diameter with the software LAS X (Software version: 3.7.4.23463, © 2020, Leica Application Suite X, Leica Microsystems, Mannheim, Germany). The average cell size was measured with one selected picture of each strain and cultivation condition. The image was split into quarters, and approximately the same number of single cells per quarter were selected to measure the size of 50 cells with the contained scale in LAS X.

### 2.9. Transmission Electron Microscopy

An amount of 10 mL of cell suspension from the last day of cultivation (day 40) was used for transmission electron microscopy (TEM). The cultures were centrifuged for 4 min at 1000× *g*. The concentrated cell suspension was transferred on a pretreated 3 mm aluminum carrier type A and closed using the flat side of carrier type B. For the pre-treatment of both carriers, a 1% solution of lecithin in chloroform was used. The samples were frozen using the high-pressure freezer EM ICE (Leica Microsystems). Under liquid nitrogen, the frozen samples were then transferred into the freeze-substitution system EM AFS2 (Leica microsystems, Vienna, Austria) with 1.5% OsO_4_ in acetone. The method for freeze-substitution was modified [31,32]. For the initial phase of substitution, the protocol was set to 90 °C for 72 h, followed by 20 °C for 24 h (warming up 5 °C per hour) and finalized at 4 °C for 18 h (3 °C per h). Three washing steps in acetone for 15 min were performed, and the samples were infiltrated afterwards in medium-hardness epoxy resin (Epoxy embedding medium, Sigma-Aldrich, Darmstadt, Germany). After 1 h, the infiltration mixtures of resin and acetone (1:2, 1:1, 2:1, 1:0) were changed, and then, the samples were left in fresh pure resin under vacuum using a desiccator and left overnight. The resin was heated at 62 °C for 48 h and cut afterwards into ultra-thin sections on Ultracut UCT ultramicrotome (Leica Microsystems, Vienna, Austria) using a diamond knife (Diatome Ltd, Nidau, Switzerland) with a 45° cutting angle. These sections were transferred onto 300 mesh copper grids. Staining was performed with solutions of lead citrate and uranyl acetate. The transmission microscope JEOL 1010 (JEOL, Tokyo, Japan) was used to observe the ultrastructure of the cyanobacterial cells by applying an accelerating voltage of 80 kV. Images were taken with a CCD camera Megaview III (Olympus, Münster, Germany).

### 2.10. Statistics

Shapiro–Wilk test was applied to test for Gaussian distribution. If the test was positive, a 2-sample *t*-test was applied to test for significance (α = 0.05). If negative, the 2-sample *t*-test was not applied.

## 3. Results and Discussion

The objective of this study was to compare the influence of different light exposure times (continuous illumination and day/night (16/8 h) cycles) on physiological aspects (growth, accumulation of PHB and glycogen, cell size, and viability) of three cyanobacteria strains (Syn.6803 (reference), Syn.192 and IFA-3).

### 3.1. Biomass Concentration

**Figure 2:**
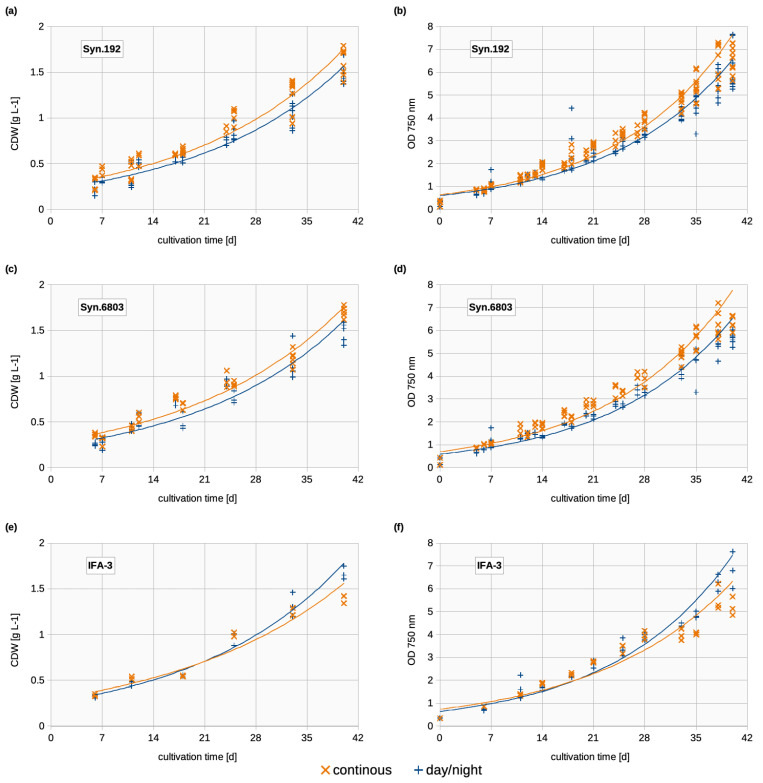
Growth over 40 days of cultivation. Plotted with an exponential trendline; Orange (x): Continuous illumination; Blue (+): Day/night cycles; (**a**) CDW [g L^-1^] of Syn.192; (**b**) OD_750_ of Syn.192; (**c**) CDW [g L^-1^] of Syn.6803; (**d**) OD_750_ of Syn.6803; (**e**) CDW [g L^-1^] of IFA-3; (**f**) OD_750_ of IFA-3.

Continuous exposure had no significant influence on the growth of *Synechocystis*, measured as cell dry weight (CDW) and optical density (OD_750_, Figure 2). However, the CDW of Syn.192 in each experiment was slightly higher under continuous illumination than under a standard day/night cycle (Figure 2a). The same was observed for Syn.6803 (Figure 2c). On the contrary, IFA-3 showed a higher CDW during day/night cultivation (Figure 2e) from sampling day 33 onwards. After 40 days of cultivation, a CDW between 1.4 and 1.8 g L^−1^ was achieved with all three strains, while no stationary growth phase was visible with either continuous light exposure or day/night cycle. All growth curves of OD_750_ and CDW show the same course. Especially in Syn.192 and Syn.6803, cell sizes increased (discussed in Section 3.4.) with continuous illumination, which affected OD_750_ and CDW. In contrast to OD_750_ and CDW, cell counting revealed higher cell densities with day/night cycles (discussed in Section 3.3.). In general, it can be seen that the selected cultivation conditions were equally suitable for all three strains, as almost the same biomass was achieved at the end of cultivation.

The achieved biomass concentrations (CDW between 1.4 and 1.8 g L^−1^) are in line with previous studies with a comparable cultivation setup (max. CDW achieved after about 40 days with Syn.192: 2.08  ±  0.04 g L^−1^, IFA-3: 1.98  ±  0.07 g L^−1^, Syn.6803: 1.85  ±  0.03 g L^−1^) [8]. The slow cell growth was mainly due to the fact that the cultivation in flasks results in a high surface layer thickness and thus relatively low light yields. Second, CO_2_ was not actively injected into the flasks, and gas exchange took place only at the surface. Experiments conducted in a pilot-scale reactor clearly show that much higher yields can be achieved with a suitable photobioreactor (Syn.192: 1 g L^−1^ in 16–20 days; 2.07 g L^−1^ in 30 days) [25,33].

### 3.2. Carbohydrate Storage Compounds

#### 3.2.1. PHB

**Figure 3:**
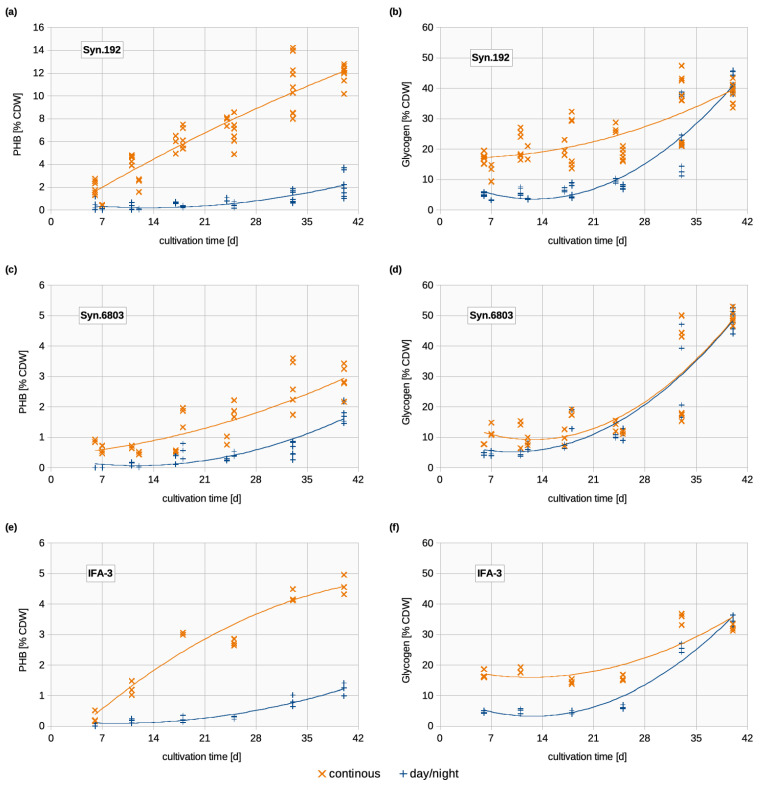
Changes in the PHB and glycogen of the three *Synechocystis* strains studied, plotted with a polynomial trendline; Orange (x): Continuous illumination; Blue (+): Day/night cycles; (**a**) PHB [% CDW] of Syn.192; (**b**) glycogen [% CDW] of Syn.192; (**c**) PHB [% CDW] of Syn.6803; (**d**) glycogen [% CDW] of Syn.6803; (**e**) PHB [% CDW] of IFA-3; (**f**) glycogen [% CDW] of IFA-3. The y-axes of PHB graphs are intentionally not in the same scaling to be able to visually recognize the change in growth associations of all strains.

Substantial differences in PHB accumulation between continuous illumination and a day/night cycle regime of 16/8 h in Syn.192 are illustrated in Figure 3a. At cultivation with day/night cycles, the PHB content remained at very low levels throughout most of the cultivation. A clear increase became visible only towards the end. The original nitrate concentration (332 mg L^−1^) decreased to less than 4 mg L^−1^ until the end of cultivation, indicating that both light regimes led to equivalent nitrogen starvation at the end of cultivation. Koch et al. (2019) [13] described that in the course of nitrogen depletion, *Synechocystis* PCC6803 underwent chlorosis (a metabolic adaption), where glycogen and PHB were increasingly accumulated and chlorophyll was degraded. Due to the degradation and metabolism of nitrogenous pigments to cope with the nitrogen deficiency, the entry into the chlorotic state is visible with a color change from bluish green to yellowish to bright orange [34,35]. This color change was also visible in the herein described experiments and indicated by decreasing chlorophyll concentrations (discussed in Section 3.2.3).

The increased PHB accumulation at continuous illumination was observed in all three strains (see also Section 3.3.). However, Syn.6803 (2.8% CDW) and IFA-3 (4.6% CDW) did not accumulate as much PHB as Syn.192 (12.0% CDW, Figure 3) and did not exceed 5% CDW. This is true with Meixner et al. (2022) [8], where Syn.192 was by far the best PHB producer with 2.7% CDW, followed by IFA-3 (2.1% CDW) and Syn.6803 (<2% CDW) under day/night cycles, which is comparable to our results at the same light regime and similar media composition. The reason for the different PHB accumulation in Syn.6803, Syn.192, and IFA-3 under the same growing conditions is not known, nor are the genetic differences between these strains, as the genomes of Syn.192 and IFA-3 have not yet been sequenced. However, the PHB genes of Syn.192 have been identified and are similar to those of Syn.6803 [36]. Only the 16S rRNA of the wild strain IFA-3 is known and matches Syn.6803 98.2% [26]. IFA-3 and Syn.6803 showed a significant difference in the amount of PHB produced after only one week of cultivation (Figure 3c,e). While PHB levels increased steadily from the beginning in continuous light exposure, the increase began much later at day/night cycles. The PHB results of all three strains tested verified that continuous illumination at around 90 µmol m^−2^ s^−1^ led to a higher accumulation of carbon storage in form of PHB in *Synechocystis*.

If a self-limiting culture medium (as in this study) is used for the cultivation of *Synechocystis* in a day/night cycle illumination regime, PHB production only starts when all the nitrogen has been used up and nitrogen limitation has occurred. This becomes clear in Troschl et al. (2018) [25] and in the results presented here, as in all three *Synechocystis* strains cultivated under day/night cycle, PHB production begins at a later point in the cultivation process than with continuous illumination (Figure 3a,c,e).

The PHB production processes are completely different with continuous light exposure. Here, PHB is already accumulated after a very short cultivation time. This seems to be typical for *Synechocystis* because all three strains behave similarly but achieve different PHB contents. Syn.192 already accumulated 2% PHB CDW after seven days of cultivation. The high PHB accumulation of this strain under continuous light exposure was expected, as it is the strongest PHB producer of the three *Synechocystis* strains investigated under classical day/night cultivation conditions. It would be interesting for further studies to investigate whether this behavior under continuous light exposure is specific to *Synechocystis* or whether it occurs in all PHB-producing cyanobacteria.

The achieved PHB accumulation in Syn.6803 (3% CDW after 40 days) at continuous illumination at 86 µmol photons m^−2^ s^−1^ is comparable with that in Wu et al. (2001) [37], where Syn.6803 accumulated 4.1% PHB CDW under continuous illumination at 4000 lux in combination with nitrogen and phosphorous starvation. This study was the only publication we found so far where continuous illumination reached comparatively high PHB amounts in Syn.6803. In most publications, higher PHB levels are obtained by applying day/night cycles instead of continuous illumination (summarized in Table 1) and are therefore in contrast to the herein described results. Reasons are most probably different cultivation conditions. Among other things, all the studies listed in Table 1 are based on a change of medium. In this study, however, a self-limiting medium was used in which the nitrogen automatically becomes a limiting factor over the cultivation period.

Since, to our knowledge, there are no studies with comparable experimental and cultivation setups (illumination intensity, self-limiting medium, etc.), the results of this study cannot be directly compared with those of other research groups. The different cultivation conditions chosen here now provide a different perspective on PHB kinetics with certain types of light exposure (continuous and day/night cycle). One possible explanation for the PHB accumulation right from the start of cultivation without nitrogen limitation is that the continuous illumination causes light stress for the cultures due to the still low cell densities in the flasks (the higher number of dead cells would also indicate stress, discussed in Section 3.4.). Another explanation could be that the cells can absorb more photons due to the continuous light exposure and therefore have more energy available, which they process in the form of PHB formation and possibly also has an effect on the cell size, which is increased with continuous light exposure (discussed in Section 3.4.). In any case, it can be said that all these theories still need to be tested in detail in further studies. From the existing data set with this experimental setup, a clear explanation for the excessively high PHB accumulation is not possible and opens up many further research possibilities.

#### 3.2.2. Glycogen

As mentioned earlier, glycogen accumulation initially increased at continuous illumination (Figure 3b,d,f) at a time when the cells had presumably not entered a chlorotic state, whereas at day/night cycles, the concentration increased with nitrogen depletion, as previously reported by Koch et al. (2019) [13]. A steep increase in all three strains in glycogen content was observed after day 25 in both cultivation regimes, indicating, moreover, that nitrogen starvation additionally induced the accumulation of glycogen and PHB. From this point on, glycogen formation in cells were decoupled from their growth kinetics.

The higher level of glycogen synthesis in the first three weeks of cultivation with continuous illumination might be explained by an increased energy supply since 7.43 mol photons m^−2^ are available for photosynthesis within 24 h with continuous illumination compared with 4.95 mol m^−2^ at a 16/8 h day/night cycle. That agrees with Velmurugan and Incharoensakdi (2018) [38], who stated in their study that there is increased glycogen accumulation with continuous illumination. Accumulated glycogen usually provides energy as carbon storage during the dark phases, which is not required to this extent by cells that are exposed to continuous light [9]. At the end of cultivation, both illumination regimes converged to approximately the same glycogen level (Figure 3b,d,f). The glycogen content in IFA-3 (Figure 3f) behaved similarly as in Syn.192 (Figure 3b). In both strains, three times more glycogen was accumulated with continuous illumination until day 25 (Syn.192: 21.1% CDW, IFA-3: 15.7% CDW, Syn.6803: 12.8% CDW). However, approximately the same glycogen level was detected on day 40 (end of cultivation) in both illumination regimes (Syn.192: 39.2% CDW and 41.2% CDW, IFA-3: 32.2% CDW and 34.3% CDW, Syn.6803: 49.3% CDW and 48.7% CDW at continuous illumination and day/night cycles, respectively). Not comparable with the curves of Syn.192 and IFA-3 are those of Syn.6803 (Figure 3d), as there was no clear difference between continuous and day/night illumination over most of the cultivation period. Especially on day 33, a high fluctuation range occurred. Glycogen content between 20% and 60% is typical for *Synechocystis* and is also in most cases higher than the PHB amount [6,13].

#### 3.2.3. Chlorophyll

**Figure 4:**
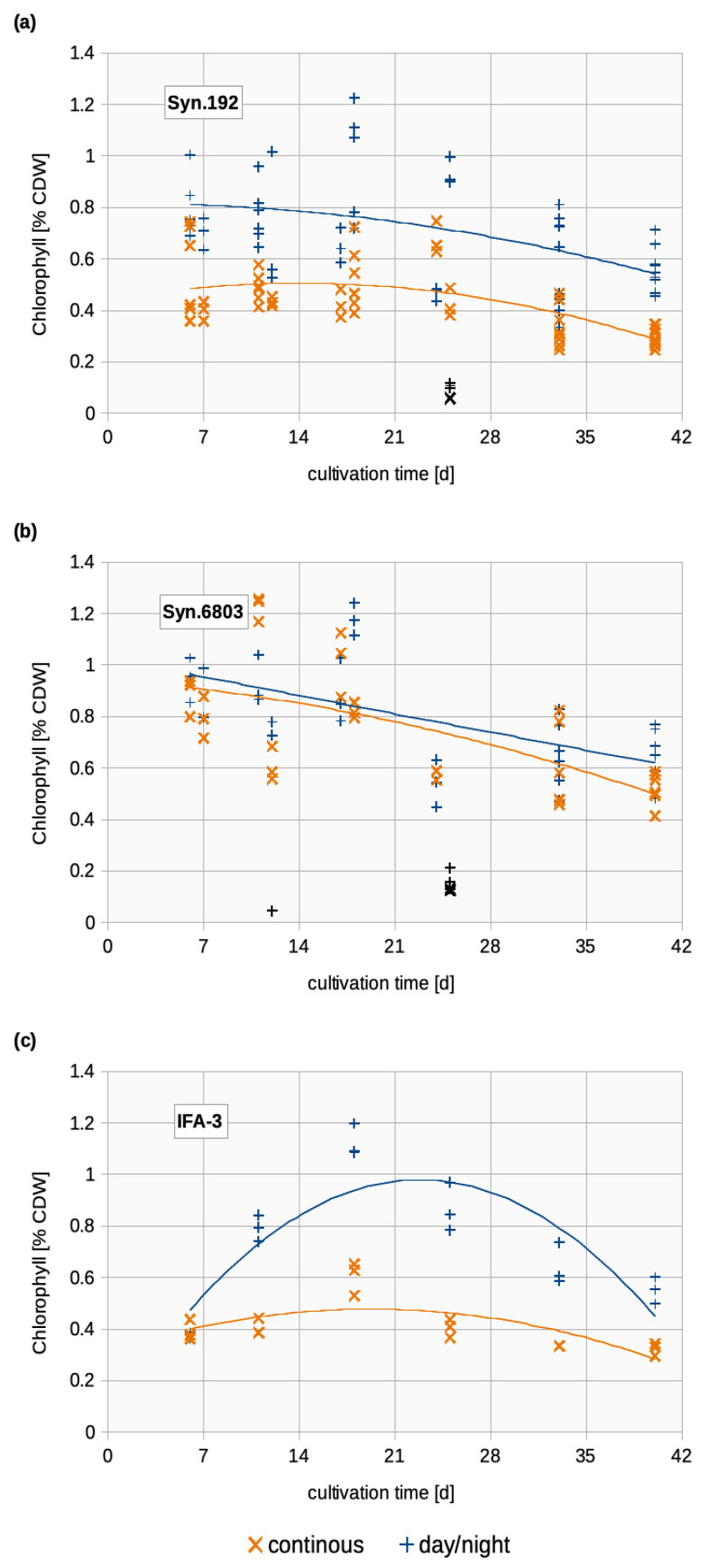
Changes in chlorophyll of the three *Synechocystis* strains studied, plotted using a polynomial trendline; Orange (x): Continuous illumination; Blue (+): Day/night cycles; (**a**) chlorophyll [% CDW] of Syn.192; (**b**) chlorophyll [% CDW] of Syn.6803; (**c**) chlorophyll [% CDW] of IFA-3.

Changes in light availability can alternate the light harvesting machinery [19]. Besides that, photosynthetic pigments are degraded by nitrogen deficiency, which is directly linked to the increased accumulation of glycogen and PHB [34]. Therefore, we aimed to establish a simple indicator of the progress of chlorosis by measuring the chlorophyll concentrations (Figure 4). The highest chlorophyll concentrations of around 1.2% CDW were achieved with day/night cycles in Syn.6803 and IFA-3. Continuous light exposure led to approximately half the amount of chlorophyll on day 40. For Syn.192 and Syn.6803, it is notable that a strong fluctuation in the values occurred at the beginning of the cultivation. This scattering became smaller in the progress of cultivation. Syn.6803 showed the smallest difference between both light regimes. The decrease in chlorophyll concentration in all strains towards the end of cultivation indicates the beginning of chlorosis [39,40]. Meixner et al. (2022) [8] reported that chlorophyll concentrations in Syn.192 did not exceed 1% CDW during cultivation and even fell below 0.5% CDW at the end. Pigment regulation is an important aspect when applying light stress or nitrogen deficiency. Muramatsu and Hihara, (2012) [41] reported that under intense light conditions (>200 µmol photons m^-2^ s^−1^), genes in photo-protective mechanisms were upregulated, while enzymes involved in pigment biosynthesis were repressed to protect the photosystems and keep the energy metabolism balanced. In their review, however, they did not mention any genes involved in PHB or glycogen synthesis. As we focused mainly on the accumulation of PHB and not on the photosynthetic machinery, we recommend future experiments to evaluate the influences on the regulation of light harvesting complexes, on the regulation of gene expression, and on the connection to PHB accumulation to enable a full understanding of the cellular energy flows.

The total amount of storage compounds was increased in Syn.192 with continuous light exposure due to an increase in PHB and constant glycogen levels. As already mentioned, the PHB accumulation at continuous illumination was far higher than that of day/night cultures. There was a significant increase already after seven days of cultivation. Even the glycogen content was significantly higher in the continuous illuminated cultures from the beginning, where the cells should not have entered nitrogen limitation. The high accumulation of carbon storage indicated an increased carbon flux towards PHB and glycogen synthesis during constant illumination compared to day/night cycles. After 40 days of cultivation, glycogen content was similar for both types of light exposure. This leads to the assumption that the PHB increase was not at the cost of glycogen decrease, instead indicating that a higher fraction of captured CO_2_ was taken up as carbon storage under continuous illumination. In both illumination regimes, the glycogen content increased steadily until day 40 of the experiments. Koch et al. (2019) [13] demonstrated that during nitrogen starvation, PHB is formed by the degradation of glycogen, which is accumulated more rapidly than PHB. However, the results of this study did not show a decrease in glycogen concentration and a simultaneous increase in PHB accumulation. A reason for this most likely is that cells in the early stage of cultivation did not undergo nitrogen starvation and were able to take up more light energy, which is then transformed into glycogen and PHB (discussed in Section 3.2.1 and Section 3.2.2.). Additionally, the chosen light condition in the form of continuous illumination is stress (as the higher number of dead cells would also indicate, discussed in Section 3.4.), for the cells and stress factors can lead to increased PHA production in bacteria, as demonstrated by Obruca et al. (2021) [14]. PHA granules help cells to cope with stress (e.g., osmotic shock, oxidative pressure, or UV radiation). An example of the physiological role of PHA is given by Slaninova et al. 2018 [42], who demonstrated that PHA granules have a UV-scattering effect. The positive effect of PHA and the survival advantage is known for many heterotrophic bacteria; therefore, it is assumed that the same mechanisms exist in cyanobacteria.

### 3.3. PHB per Cell

**Figure 5:**
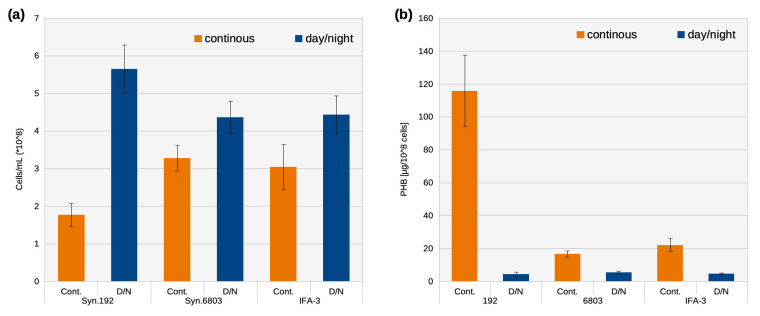
Cell numbers (**a**) and PHB-values calculated to equal cell numbers (10^8^ cells) (**b**) after 40 days of cultivation under continuous light exposure (orange) and day/night (16/8 h) cycles (blue) of Syn.192, Syn.6803, and IFA-3.

The different PHB content of all three strains in the two illumination regimes is even more clearly visible if the PHB content is related to the same cell numbers (Figure 5). By far the most PHB per cell (110 µg/10^8^ cells) was stored in Syn.192 with continuous exposure after 40 days of cultivation. In Syn.6803 and IFA-3, continuous exposure resulted in values between 15 and 25 µg/10^8^ cells. A 16/8 h day/night cycle resulted in a similar PHB content on all three strains (about 5 µg/10^8^ cells).

**Figure 6:**
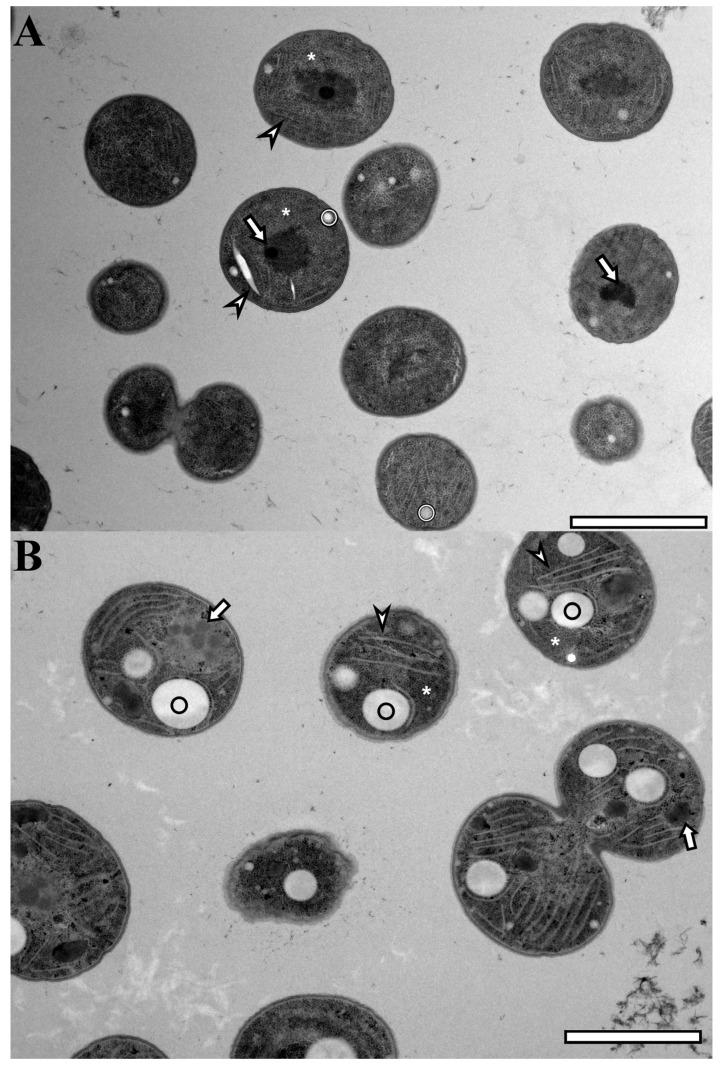
TEM pictures of Syn.192 cultivated with day/night cycles (**A**) and continuous illumination (**B**). Circle (◯): PHA granule, arrowhead (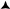): Thylakoid membranes, arrow (⇧): Carboxysome, star (∗): Glycogen granules; Scale bar 2 μm.

This substantial difference in increased PHB accumulation is also illustrated in TEM pictures (Figure 6) of Syn.192, as the whole PHB granules increased in size. Different causes for this increase are possible. It might be derived from the higher energy input (7.43 mol photons m^−2^ compared to 4.95 mol m^−2^ within 24 h, discussed in Section 3.2.1 and Section 3.2.2) or/and as a physiological reaction to the missing dark cycles, resulting in light stress. Slaninova et al. described in 2018 [42] a light scattering effect on PHB granules in *Cupriavidus necator* (Bacteria) to protect cells from damage caused by UV irradiation. Such a scattering effect is rather unlikely due to the significantly lower PHB content in *Synechocystis*.

### 3.4. Cell Size and Viability

**Figure 7:**
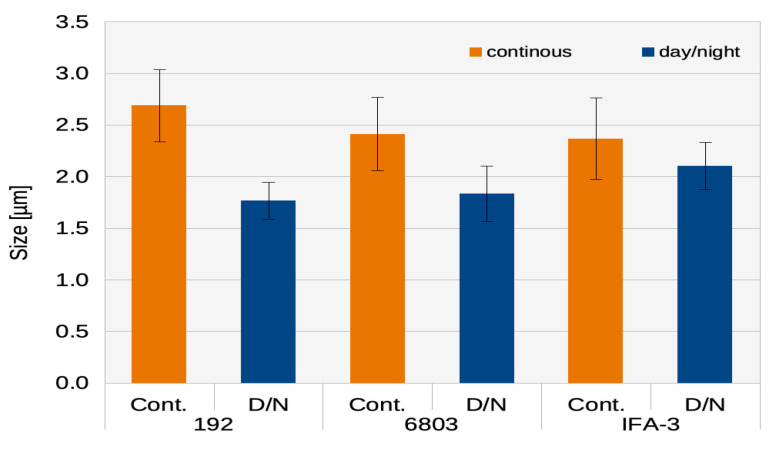
Cell size distribution during continuous light exposure (orange) and day/night cycle (blue) of Syn.192; Syn.6803; IFA-3;.

Although both CDW and OD_750_ were approximately the same, day/night cultivation resulted in a higher cell count compared to continuous exposure in all three strains but was especially pronounced in Syn.192 (Figure 5a). The cell size of IFA-3 was not significantly increased (Figure 7), and OD_750_ and CDW measurements showed better growth with day/night cultivation (Figure 2e,f).

In this study, a distinct difference in cell size was observed especially in Syn.192 (Figure 7). In general, there is little volumetric variance in the cell size of different cell types despite fluctuations during the cell cycle and the influences of external stress factors. This strict control, which follows generic linear scaling relations between cell volume, CDW, and the volume of the nucleus, shows how important cell size is for monitoring cell functions [43]. Nevertheless, several influencing factors can also affect cell sizes and morphology, like nutrient availability and different properties of light in photosynthetic organisms [19]. Kellogg and Levin (2022) [44] reviewed that nutrients can influence cell size as external modulators through changes in cell growth and cell cycle progression. For example, it was found that *Escherichia coli* increased its cell size threefold when cultured in nutrient-rich media than in nutrient-poor conditions. Considering that illumination determines the photosynthetic activity and therefore also the CO_2_ fixation rate, it can be suggested that continuous light, when compared to day/night illumination, can be referred to as a carbon-rich condition. Koch et al. (2020) [22] genetically modified Syn.6803 and achieved a maximum PHB content of 81% CDW but observed no change in cell size. Thus, a PHB content of 12% CDW, in our case, could not be the reason for the increased cell size but rather the increased energy supply (7.43 mol photons m^−2^ at continuous illumination compared to 4.95 mol m^−2^ at a 16/8 h day/night cycle within 24 h, discussed in Section 3.2.1 and Section 3.2.2). An increase in cell size during continuous illumination was not mentioned by the authors summarized in Table 1.

Syn.6803 has typically a cell size of 1.5 µm [45], which was also observed in our study under day/night cycles. Osanai et al. (2013) [45] reported an increase in cell size to approximately 2.52 µm of Syn. 6803 by the overexpression of sigE, a transcriptional regulator that is induced when nitrogen is depleted and is involved in glycogen catabolism, glycolysis, and the oxidative pentose phosphate pathway. The overexpression of sigE also enhances PHB production. In this study, approximately the same cell sizes as in the study by Osanai et al. (2013) [45] were found (Figure 7). The measurement of expression levels was not the intention of this study. Therefore, further studies should look at whether sigE is overexpressed by continuous illumination. This overexpression could be the reason/trigger for the increased PHB production. 

Besides cell size, cell viability was measured. For this purpose, samples were immediately transported to TU Brno, where 10,000 cells of Syn.192 (as a high PHB producer) and Syn.6803 (as reference) were counted. A higher fraction of dead cells occurred under continuous illumination than during day/night cycles (Table 2).

The increased cell density throughout cultivation led to the self-shadowing of the culture at some point. This means that the cells were repeatedly subjected to short dark phases due to the turbulent flow of the culture, and photoinhibition occurs only at the surface of the culture. In order to come close to continuous exposure in a possible realization on a production scale, the culture must be flooded with light (e.g., with a low layer thickness). Cultivation systems with low layer thicknesses are, for example, flat-plate PBR [46] or thin-layer cascades [47,48].

A flashing light effect in the form of short illumination phases has been proven to be a promising microalgal cultivation system because it leads to optimal integrated light doses for photosynthesis and growth without cells undergoing photoinhibition [49]. As the cells still experienced a repeating but random change between illumination and dark phases in short periods, even under continuous illumination, the regeneration of the photosystems was enabled. This makes it difficult to give a clear conclusion about whether the cells were stressed due to continuous exposure or due to overall energy input. However, since the percentage of dead cells was higher with continuous light, we suggest that this form of illumination is indeed a form of stress for the cells (PHB accumulation right at the start of cultivation without nitrogen limitation could also indicate stress, discussed in Section 3.2.1).

Troschl et al. already showed in 2018 that under good cultivation conditions and day/night cycles, at least 12.5% PHB CDW is possible with Syn.192 in a pilot scale photobioreactor [25]. An even higher PHB content in Syn.192 might be achieved under continuous illumination.

## 4. Conclusions

Continuous illumination at 86 µmol m^−2^ s^−1^ led to physiological changes in all three *Synechocystis* strains. Only minor differences in growth were observed based on OD_750_ and CDW analyses, but a significant reduction in cell number was found under continuous light exposure. Increased cell size during continuous illumination, most pronounced in Syn.192, was the explanation for the apparent contradiction, a finding that was not described in the literature so far. PHB and glycogen content were increased with continuous illumination in all strains before nitrogen limitation occurred. The differences in PHB contents increased even further for Syn.192 and IFA-3 throughout cultivation. The highest PHB values with up to 14.2% CDW were obtained with Syn.192 and continuous light exposure. The least difference in PHB and glycogen accumulation between both light regimes was observed in Syn.6803, which is the most studied cyanobacterial strain. Our results, in combination with further research, will contribute to a better understanding of the physiological roles of PHB in cyanobacteria and can increase the chances for PHB production by phototrophic biotechnology.

This study focused mainly on the evolution of carbon storage over the course of cultivation. For further studies, analyses on gene expression patterns or ATP and NADPH content could give more detailed insight into the metabolic response of *Synechocystis* to continuous light exposure.

## Figures and Tables

**Table 1:** The results of a literature search of continuous light versus light/dark cycle and its effect on PHB accumulation.

Strain	Effect of Continuous Illumination	Reference
*Synechocystis* PCC 6714	No significant difference in PHB accumulation between continuous illumination and day/night cycle light regimes	[21]
*Synechocystis* PCC 6803	Higher PHB content under day/night cycles compared to continuous illumination	[20]
*Synechocystis* PCC 6803	No significant difference between continuous and alternating illumination	[22]
*Synechocystis* PCC 6803	4.5% PHB at day/night cycles compared to 2.4% PHB at continuous illumination	[23]
*Desmonostoc muscorum* (formerly *Nostoc muscorum*)	Higher PHB content at day/night illumination (7.63%) compared to 3.31% with continuous illumination	[24]

**Table 2:** Amount of dead cells of 3 flasks of Syn.192 and Syn.6803 at continuous illumination (Cont.) and day/night cycles (D/N), measured with flow cytometry.

Strain	Light	Dead Cells (%)
Syn.192	Cont.	20.61 ± 0.88
	D/N	14.62 ± 1.77
Syn.6803	Cont.	24.38 ± 0.20
	D/N	14.46 ± 2.51

## Data Availability

The original contributions presented in the study are included in the article, further inquiries can be directed to the corresponding author.
